# Identification of dyes and matrices for dye doped polymer waveguide emitters covering the visible spectrum

**DOI:** 10.1038/s41598-022-10145-8

**Published:** 2022-04-12

**Authors:** L. F. Paz, M. Caño-García, M. A. Geday, J. M. Otón, X. Quintana

**Affiliations:** CEMDATIC, ETSIT Telecomunicación, Universidad Politécnica de Madrid, Av. Complutense 30, 28040 Madrid, Spain

**Keywords:** Lasers, LEDs and light sources, Optical materials and structures, Optical techniques, Other photonics, Materials science, Nanoscience and technology

## Abstract

Polymer based photonic devices offer the possibility cost effective roll-to-roll manufacture of photonic devices. The incorporation of luminescent dopants within a solid polymer waveguide allows for the generation of light within the device avoiding tedious mechanical light coupling. However, when a dopant is embedded in a solid matrix, depending on its concentration and the nature of materials involved, the emitted light may be quenched due to aggregation effects. In this work, thin films and ridge waveguides processed by UV-photolithography have been successfully obtained from a selection of standard photopolymerizable organic monomers, SU8, EpoCore and OrmoStamp doped with a selection of standard dyes like Rhodamine-B, Coumarin-540A and Pyrromethene-580. All structures were manufactured on glass substrates. An analysis of the solubility and optical properties including band gap energy, absorption coefficient ($\alpha$) and fluorescence of the doped photoresists at different concentrations has been performed. Photoresists doped with Rhodamine-B shows a higher energy of indirect allowed band gap transition (2.04–2.09 eV) compared to previously reported pure Rhodamine-B thin films (1.95–1.98 eV). Fabrication protocols of dye doped photoresists covering the entire visible spectrum is established.

## Introduction

In recent years, photonic integrated circuits (PICs) based on polymeric materials have gained increasing attention by scientific community for an ever widening range of applications including optoelectronics^1^, sensors^2,3^, lighting^4^ and optical computing^5^.

Manufacturing PICs on a polymer platform rather than the more conventional and compact silicon platforms has two major advantages: Polymers may be transparent in the visible wavelength spectrum, which means that dyes employed in conventional microscopy can be employed, and a polymer platform allows for future translation to cost-effective roll-to-roll manufacture of the final photonic circuit^6^.

The incorporation of luminescent materials such as quantum dots^7^, dyes^8^ or photoluminescent copolymers^9^ into polymeric host matrix enables the generation of organic light emitters with applications in fields as solar cells^10^, optical amplifiers^11^ and gas and pH sensors^12,13^.

Dyes covering fully the visible range of the spectrum have been used in polymeric matrices showing solubility and stability, although depending on the dye and matrix nature, processes as Aggregation Caused Quenching (ACQ)^14^, Aggregation-Induced Emission (AIE)^15^ and Aggregation Enhanced Emission (AEE)^16^ might take place affecting the emission properties. The complexity of the matrix-dye interactions makes it difficult to predict what matrix to combine with what dye to achieve a waveguide emitting at a given wavelength.

In this work, the epoxy-based polymers EpoCore and SU8 and inorganic–organic hybrid material OrmoStamp have been studied as polymer matrices doped with dyes as Rhodamine-B (RhB), Coumarin-540A (C540A), Cibacron-Yellow (CBY), Fluorescein (FL), Pyrromethene-580 (Py580) and Red light-emitting spiro copolymer (RLSC). A much wider variety of dyes have been tested but has been discarded due to limited solubility and/or visible indications of aggregation as shown in the Supplementary material.

The photosensitive nature of these resins, allow for micro/nano fabrication techniques as UV-lithography^17^, direct laser writing^18^, e-beam^19^, ink-jet^20^ and UV-nanoimprint^21^. The dyes employed in this work cover the visible range of the spectrum, targeting future applications such as integrated lasers, biosensors, and optical communications.

The optical properties of dye doped photoresists waveguides processed by UV-photolithography are studied within the achieved range of concentrations. The protocols of fabrications of each doping concentration and resist are presented and discussed.

To the best of our knowledge, waveguides of doped OrmoStamp processed by UV-lithography hasn’t been reported previously, although it has been employed as cladding material^22^.

The motivation for this work, is the generation of polymer luminescent matrices, compatible with molding into waveguide structures, that are easily combined with conventional waveguide-based sensors. This work forms the initial step towards photonic waveguide-based sensors, that don’t require tedious and mechanically challenging coupling of an external light, typically laser, light source. The luminescent matrix may be excited by flooding with either external laser light or even light emitting diodes, without the need for careful coupling of light into a guided mode in the micro-meter sized waveguide structure.

Identifying matrix and dye combinations that covers the full visual spectrum permits a sensing platform, with full spectral freedom that can be optimized to a wide variety secondary dyes for direct color detection and/or excitation, that may be employed in a final waveguide-based sensor. The waveguide sensor, will not typically be made entirely out of the luminescent structure, since this structure will tend to be absorbing where it is not excited by the light flooding, and the quality of the emitter as a conventional waveguide becomes secondary.

## Methods

### Materials

Five dyes have been studied: Rhodamine-B (RhB), Fluorescein sodium salt (FL), Cibacron Brillian Yellow 3G-P (CBY), Coumarine-540A (C540A) and Pyrromethene-580 (Py580) with molecular weight of 479.01, 376.27, 831.02, 309.29 and 374.32 g/mol respectively, as well as a photoluminescent Red Light-Emitting Spyro Copolymer (RLSC) with an average molecular weight of 180,000 g/mol. All dyes were supplied by Sigma Aldrich except to C540A and Py580 that has been provided by Luxottica Exciton. The photoresists used for the UV-photolithography and thin films fabrication were the series of EpoCore-2 and SU8-2001 as epoxy-based negative photoresists and the organic–inorganic hybrid negative photoresist, OrmoStamp. All photoresists were supplied by MicroChem. Glass substrates were used as manufacturing support for the samples.

### Samples preparation and characterization

The photoresists and dyes were used as purchased. The prepolymer of SU8 and EpoCore are solids at room temperature and come dissolved in a polymer specific solvent. In the case of OrmoStamp, the prepolymer is liquid at ambient temperature and it is solvent free.

The UV-photolithography thin film and microstructure manufacturing protocols are described below. The dye is dissolved in the photoresist’s solution directly, with no added solvents. In the case of OrmoStamp, the prepolymer acts also as solvent for the dyes. It was not possible to obtain samples of EpoCore doped with Py580 due to its very low solubility.

To obtain homogenous solutions, vigorous magnetic stirring during 1 h followed by ultrasonic bath of 2 h were applied for every prepolymer dye doped solutions before film deposition, the substrates were treated with deep UV cleaner during 20 min. in a UVO-Cleaner (Jelight Company, Model 18) to promote the adherence. The thin film deposition was performed by spin coating (Laurell, WS-650-23NPP). The waveguides structures were obtained using UV-photolithography with a power of 4 mW/cm^2^ (OAI, Hybralign Series 400).

#### SU8 dye doped microstructures preparation

Prepolymer solutions of 1 mL of RhB and Py580 at different concentrations were prepared. In the case of SU8 doped with RhB the working concentrations were 1, 4 and 7 mM and 1, 4, 7 and 20 mM for Py580 doping. Once the cleaned and surface activated glass substrate is placed on the spinner, the homogeneous solution is dispensed with a Pasteur pipette, trying to cover all the glass surface avoiding bubbles. The spinning parameters were 2000 rpm during 30 s for every sample. After thin film deposition, the samples are placed on a hot plate to complete the two steps prebake: 65 °C during 3 min and 95 °C for 6 min. Subsequently, the samples are exposed to UV light using a photomask with 15 µm wide waveguides and 160 µm pitch. The exposition time depends on dye doping. For the case of RhB at 1, 4 and 7 mM the exposure times were 250, 1400 and 1700s, and for Py580 at 1, 4, 7 and 20 mM the required times were 100, 150, 250 and 650 s. Then, a two steps post bake is performed: 65 °C during 3 min and 95 °C for 10 min. To remove the prepolymer that has not been exposed under UV light, a development step of 1 min is required. The solvent used in this step is 1-Methoxy-2-propyl acetate. Finally, the process is completed by a hardbake where samples are placed into oven at 150 °C during 30 min.

The manufacturing protocol between different concentrations of RhB and Py580 doping, differs in the exposition times. The exposition times have been optimized in all samples to get polymerized structures and increases with the dye concentration. The dye doping within the matrix compromises the chemical reactions in the polymerization and absorbs UV light during the photoinitiation.

#### EpoCore dye doped microstructures preparation

Prepolymer solutions of 1 mL of EpoCore doped with RhB at concentrations of 1, 3 and 5 mM were prepared. The same general SU8 manufacturing protocols are also followed, including the same photomask used, but with slight differences. The spin coating deposition was done at 3000 rpm for 30 s. The prebake is done in two steps but at 50 °C during 3 min and 90 °C for 6 min. The exposition time required for 1, 3 and 5 mM of doping were 70, 150 and 250 s. The postbake step is 65 °C during 3 min followed by 95 °C for 10 min. To remove the unexposed prepolymer, the development is done using commercial developer (mr-Dev 600, supplied by MicroChem). Finally, the hardbake is at 140 °C during 30 min.

Like in the case of SU8, the exposition times also increase with the dye concentration. The changes in exposition times between SU8 and EpoCore are not comparative which we attribute the differences to chemical structures of the compounds and the photoinitiator.

#### OrmoStamp dye doped microstructures preparation

Solutions of 3 mL of OrmoStamp doped with RhB and C540A at 2, 5, and 7 mM and 10, 30 and 50 mM were prepared respectively. Similar protocol manufacturing than previous materials is followed. The photomask used in the exposition was a single waveguide with a width of 1000 µm. To get fully homogenous prepolymer solutions, the stirring time is increased up to 5 h followed by ultrasonic bath of 3 h. The spin coating deposition is done at 3000 rpm during 30 s. The prebake of every sample is 80 °C during 2 min. As OrmoStamp is solvent free, this step is only required to increase adherence. The UV exposure times depends on dye content. For the case of RhB at 2, 5 and 7 mM, the exposition time were 1500, 2000 and 2500 s respectively and for C540A doping at 10, 30, 50 mM the required time were 2000, 2400 and 2600 s. The post bake is a single step of 130 °C during 10 min. The developer used was OrmoDev (supplied by MicroChem) during 1 min. Finally, a hard bake is carried out at 130 °C during 30 min.

Like in the previous cases, the exposure times of OrmoStamp also increases with dye concentration for the same reasons.

The thickness of the EpoCore and SU8 structures are, 2 and 1.2 µm, respectively. For the case of OrmoStamp, the thickness obtained was 4.4 µm. Non-contact photo lithography was used for the OrmoStamp waveguide lithography due to the liquid nature of the prepolymer, and consequently wider waveguide structures had to be employed.

### Dimensional and morphologic characterization

The thicknesses (height) were measured by profilometry in films using mechanical marking with a plastic spatula an on the ridge waveguides structures (Veeco, Dektak 150). 3D maps of the surface topography were likewise done in the profilometer. The widths of the waveguides were estimated optically in a standard polarizing microscope, and confirmed in the profilometer.

### Optical characterization

**Figure 1:**
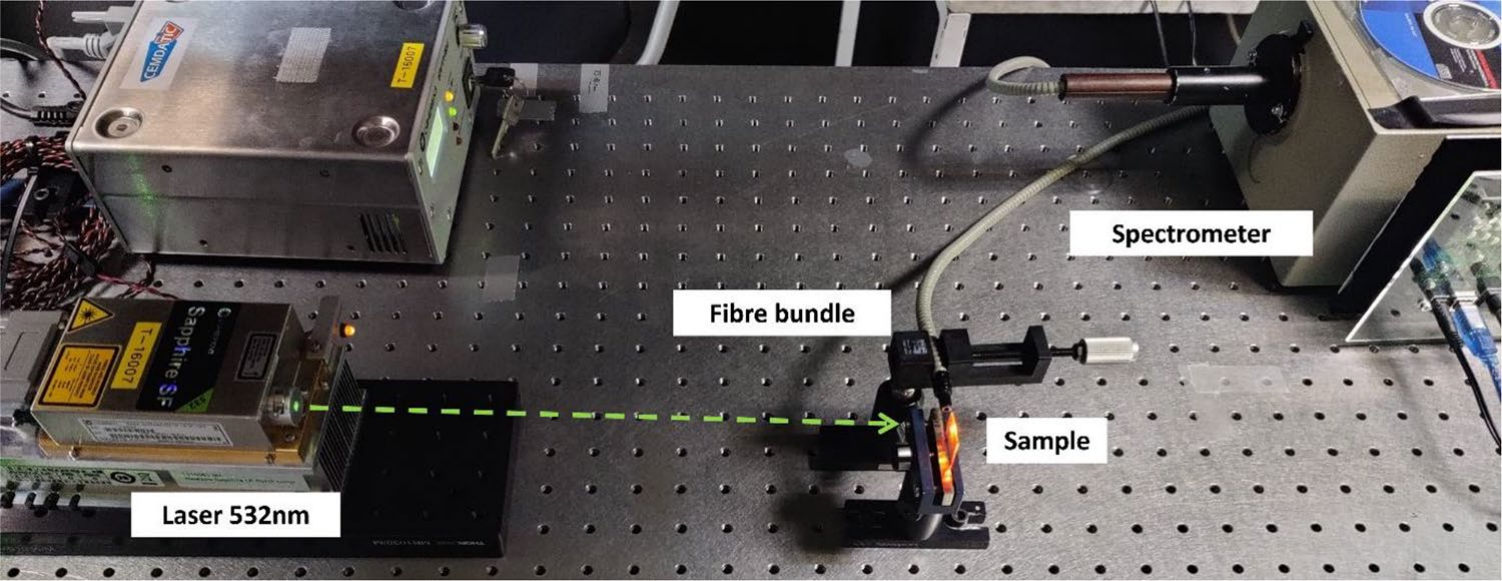
Photograph of setup used for fluorescence characterization of dye doped photoresists. A 532 nm laser impinges perpendicularly to the sample, and the fluorescence is measured edge on using a fiber bundle and a spectrophotometer*.*

The optical absorption and emission characterization was performed on the films deposited on glass substrates, rather than on the developed waveguides to ensure a more homogenous excitation of the dye inside the polymer matrix. The absorption spectra were measured using UV–VIS spectrophotometer (Perkin-Elmer, Lambda 2) whereas the fluorescence emission was measured exciting the samples perpendicularly to the film surface. The edge emission from the samples was collected by a multimode fiber and analyzed with a CCD sensor (Hamamatsu, S11155-3048-02) mounted in a custom-made spectrum analyzer. A schematic setup of the emission characterization is shown in Fig. 1. Photograph of setup used for fluorescence characterization of dye doped photoresists. A 532 nm laser impinges perpendicularly to the sample, and the fluorescence is measured edge on using a fiber bundle and a spectrophotometer. The pumping sources were lasers at 532 nm, for photo-resins doped with RhB and Py580, and 405 nm for photopolymers doped with C540A.

## Results

### Manufacturing

The maximal dye concentration in the various prepolymer solutions has been determined. Table 1 summarizes the solubility and polymerization properties for the three photoresists and six dyes studied.

**Table 1:** Maximum dye concentrations (M), limited by solubility and polymerization inhibition.

	RhB	Py580	C540A	FL	CBY	RLSC
SU8	1200 × 10^–5^ ± 2 × 10^–5^	3000 × 10^–5^ ± 2 × 10^–5^	^‡^	^†^	^†^	^†^
EpoCore	700 × 10^–5^ ± 2 × 10^–5^	^†^	^‡^	^†^	^†^	^†^
OrmoStamp	500 × 10^–5^ ± 2 × 10^–5^	^†^	10,000 × 10^–5^ ± 2 × 10^–5^	^†^	^†^	^†^

^†^Limited by solubility.

^‡^limited by polymerization inhibition.

The thin films and ridge waveguides manufacturing of doped photoresists is limited not only by the dye solubility within the prepolymer solution itself, but also by dye inhibition of the photo-initiation and subsequent cross-linking during post bake step. Finally, an important aspect is the light emission properties of the dye in polymerized photoresist.

The dye presence causes not only UV-light absorption, but the dye molecules may interfere with the chemical interaction between the growing prepolymer chains. The polymerization inhibition limited the RhB doped SU8 microstructures to concentrations to below 7 mM even when the UV doses were increased tenfold, leading to poorly/undefined microstructures at the end of the photolithographic process. The same effect limited the SU8 doping with Py580, being 20 mM the highest doping concentration that allowed to obtain well defined microstructures. On the contrary, the limitation imposed to OrmoStamp and EpoCore both doped with RhB, was the dye solubility as it was not possible to obtain homogenous solution with higher concentration than 7 and 5 mM, respectively.

**Figure 2:**
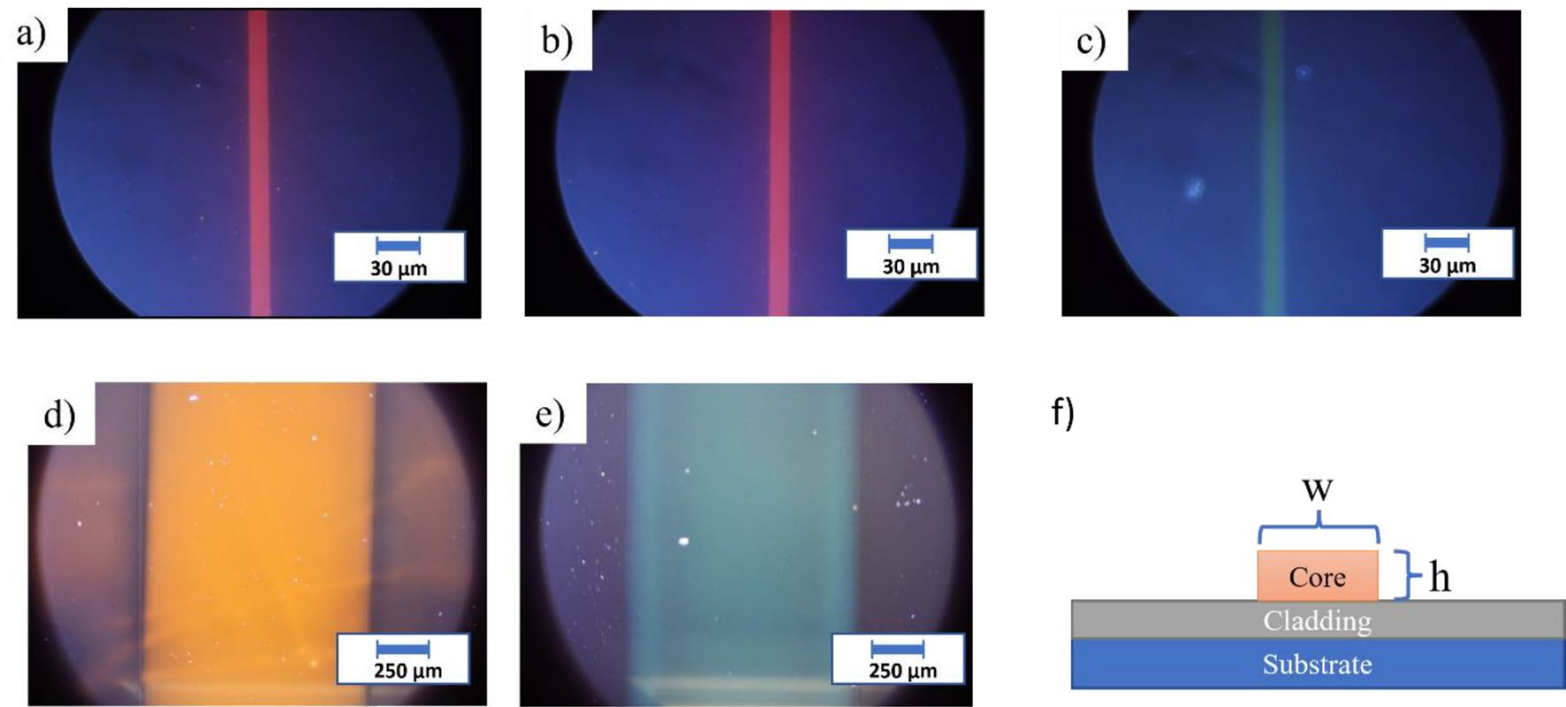
Ridge waveguides of (**a**) SU8 doped with RhB at 7 mM, (**b**) EpoCore doped with RhB at 5 mM, (**c**) SU8 doped with Py580 at 5 mM, (**d**) OrmoStamp doped with RhB at 7 mM, (**e**) OrmoStamp doped with C540A at 50 mM and (**f**) Structure of a ridge waveguide were *w* and *h* correspond to the width and height, respectively. The width of a-c waveguides is 15 µm whereas the OrmoStamp is 1100 µm.

In SU8 15 µm wide waveguides with 160 µm pitch were manufactured using hard contact UV-photolithography, as mentioned above. Figure 2 shows SU8 waveguides dye doped with RhB and Py580, confirming that the presence of dye does not affect the waveguide manufacturing. Similar results were achieved in OrmoStamp and Epocore, the employed dye concentrations did not affect the final waveguide quality in any appreciable manner although the UV exposure time in had to be increased significantly upon doping as indicated above.

Table 2 summarizes the structural properties of waveguides that have been manufactured. No changes in the thickness were experienced by a photoresist doped with distinct dye. The waveguides of OrmoStamp were made wider to avoid contact lithography. The height of the spin-coated films was identical in both film and developed waveguides.

**Table 2:** Dye doped waveguide dimensions.

Photoresist	Dye	w	h
SU8	RhB	15 ± 2 µm	1.2 ± 0.1 µm
SU8	Py580	15 ± 2 µm	1.2 ± 0.1 µm
EpoCore	RhB	15 ± 2 µm	2.0 ± 0.1 µm
OrmoStamp	RhB	1100 ± 20 µm	4.4 ± 0.1 µm
OrmoStamp	C540A	1100 ± 20 µm	4.4 ± 0.1 µm

Topographies of the film morphology can be found in the supplementary material.

### Absorption characterization

The absorbance (A) of a material depends on the absorption coefficient and the path length measured.

1 $$I={I}_{0}\cdot {e}^{-\alpha \cdot d}={10}^{-A}$$

where α corresponds to absorption coefficient, d is the film thickness and $I$ and ${I}_{0}$ are the output and input light intensity, respectively.

**Figure 3:**
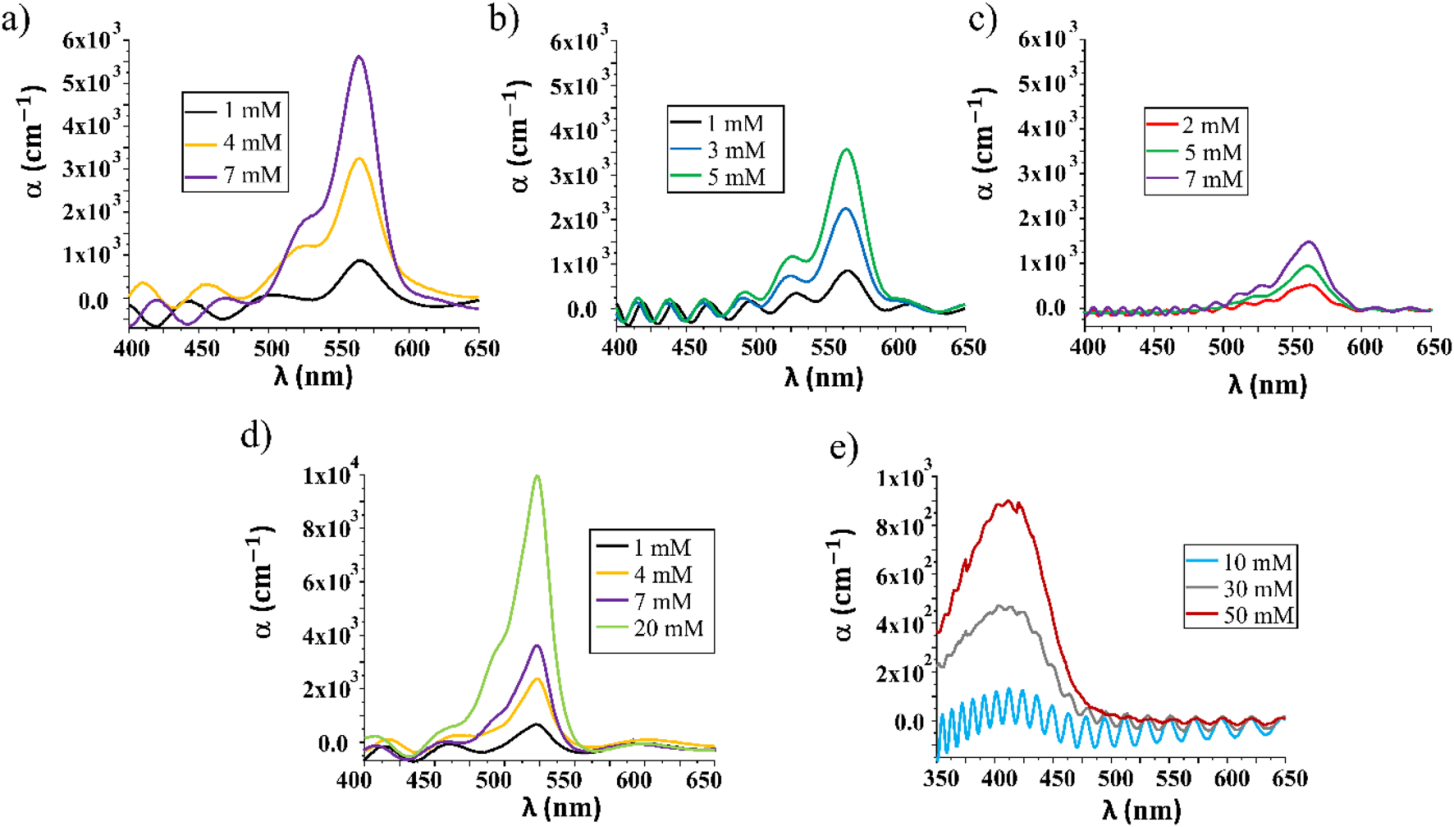
Spectral distribution of absorption coefficient (α) curve of SU8, EpoCore and OrmoStamp thin films (**a**) SU8 doped with RhB at concentrations of 1 mM, 4 mM and 7 mM, with 1.2 µm of thickness, (**b**) EpoCore doped with RhB at concentrations of 1 mM, 3 mM and 5 mM, with 2 µm of thickness, (**c**) OrmoStamp doped with RhB at 2, 5 and 7 mM of concentration, with 4.4 µm of thickness, (**d**) SU8 doped with Py580 at 1 mM, 4 mM, 7 mM and 20 mM, with 1.2 µm of thickness, and (**e**) OrmoStamp doped with C540A at 10 mM, 30 mM and 50 mM with 4.4 µm of thickness. All concentrations reflect the dye concentration in the prepolymer.

The absorption coefficient (α) vs. wavelength of 1.2 µm thin films of thickness, made from by SU8 with RhB and with Py580 respectively for different doping concentrations is shown in Fig. 3a,d. The former shows the principal absorption peak at 565 nm with a shoulder at 525 nm. In the latter, the main absorption peak is centered at 523 nm with a shoulder that appears at higher energy at 494 nm.

Figure 3b shows the spectral absorption coefficient of a set of thin films of EpoCore photoresist with a thickness of 2 µm, doped with RhB at three concentrations: 1, 3 and 5 mM. Like in SU8 the absorption peak is situated around 565 nm.

A Fabry-Pérot etalon interference pattern can be observed in absorption measurement caused by the reflections back and forth in the thin film. The changes in absorption of the same molar solution of dye in the prepolymer solution reflects principally the volume change in the materials. The Ormostamp (not having any solvent) practically maintains its volume upon polymerization, while both SU and EpoCore will reduce their volume, as the solvent evaporates during pre-bake, leading to a much higher dye concentration in the final polymer. The Fabry–Perot interference oscillations illustrate the thickness uniformity in the different samples. In samples with high absorption the oscillation amplitude is attenuated as is clearly visible in Fig. 3e.

The presence of the interference pattern leads to the appearance of a shoulder in several of the absorption spectra.

The absorption spectra of thin films of SU8 and EpoCore doped with C540A shows no specific absorption at any range of concentration limited by its solubility (Supplementary Fig. S1). The prepolymer solution with dye, is visibly absorbent (Supplementary Fig. S2), as is the thin films prior to post-baking, however, after the post bake the film loses its colour. We attribute this to either partial phase separation leading to molecular orbital (π-π) interactions between the dye molecules as seen in high solvent or matrix concentrations^23^ or that the dye disintegrates during the matrix polymerization, or couples to the polymerized matrix in a way that drastically changes the absorption spectra.

As can be seen from Table 1, RhB was soluble in all three matrices. Figure 3 shows the spectral distribution of absorption coefficient (α) of thin films of OrmoStamp c) doped with RhB at 2, 5 and 7 mM of concentration and e) doped with C540A at 10, 30 and 50 mM of concentration with 4.4 µm of thickness. Considering the RhB doping, the main absorption peak appears at 561 nm, and the shoulder at 520 nm, *i.e.* both blue-shifted approximately 5 nm with respect to the epoxy based photoresists doped with the same dye.

Unlike in the epoxy-based resins C540A is readily dissolved in OrmoStamp, and an absorption peak appears centered at 412 nm with concentrations up to 50 mM. Relatively thick 4.4 µm thin films were produced.

Very low absorption coefficients were obtained for thin films of OrmoStamp doped with RhB compared to doped epoxy base photoresists.

Smooth absorption signal is obtained for 10 Mm of C540A content, where Fabry-Pérot interference is clearly predominant.

The maximum of absorption coefficients is approximately linearly independent of the dye concentration in each of the matrices separately. The difference in absorption for a given nominal concentration, reflects that the concentrations are defined in the prepolymer mixture, which contain different amounts of solvent, leading to different final concentrations in the polymerized structures.

### Emission characterization

**Figure 4:**
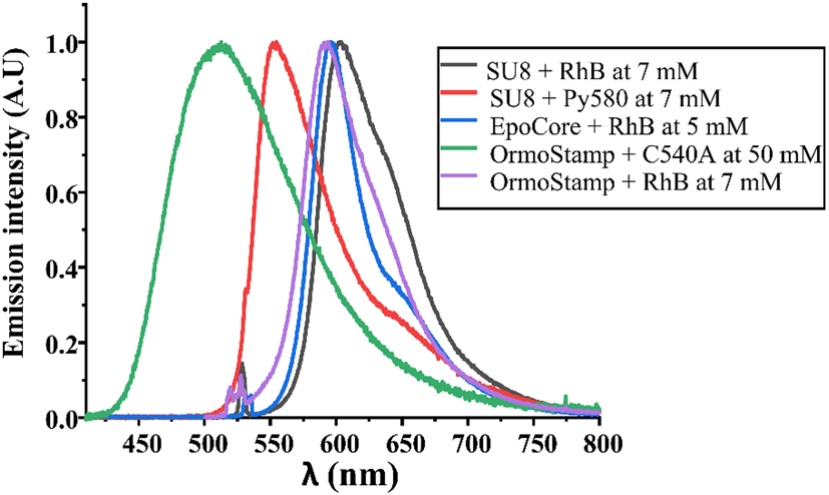
Emission spectra of dye doped thin films.

Emission spectra of thin polymeric films of SU8, EpoCore and OrmoStamp doped with RhB, Py580 and C540A are shown in Fig. 4. The combination of the three dyes and a suitable matrix cover the entire visible spectrum which was the aim of this study.

The same films as above with thicknesses of 1.8 µm, 2 µm and 4.4 µm, respectively were characterized. The polymers doped with RhB and Py580 were excited with a green 532 nm laser while the C540A doping was excited with blue laser at 405 nm. OrmoStamp doped with C540A shows the broadest band emission with a FWHM of 112 nm.

### Band gap characterization

Tauc et al.^24^ have developed a method for calculating the band gap energy of amorphous semiconductors, which has proven applicable to dye-doped polymers^25,26^. The relation between absorption coefficient, *α*, and photon energy relation can be expressed as follows:

2 $$\alpha = \frac{B\cdot  {(h\nu -{E}_{gap})}^{m}}{h\nu }$$

where *B* is a constant related to the transitions’ probability, *h* $\nu$ is the photon’s energy, and *E*_*gap*_ is the band gap energy. The value of m depends on the considered transition: for direct-gap allowed and forbidden transitions, m = 1/2 and m = 3/2, respectively, and for indirect-gap allowed and forbidden transitions, m = 2 and m = 3, respectively.

**Figure 5:**
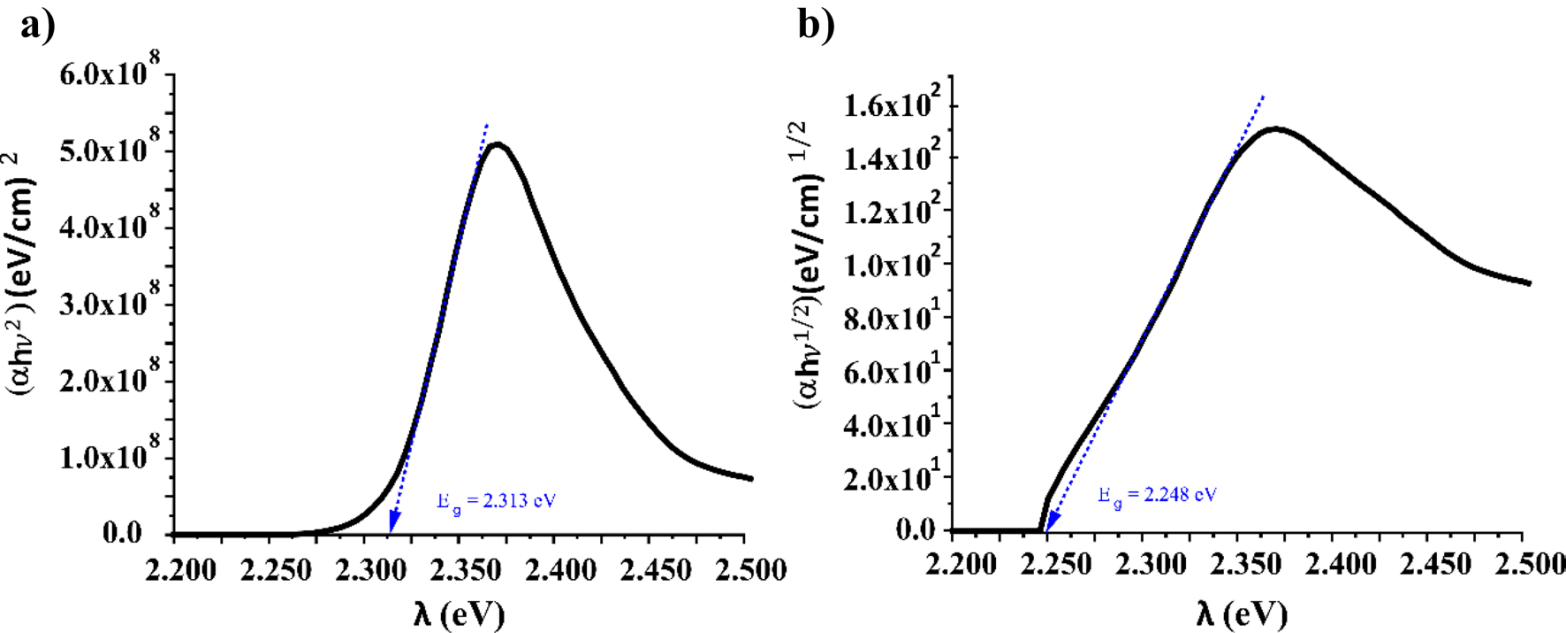
Relationship between (αhν)1/m (eV/cm)1/m and Energy (eV) of thin films of SU8 doped with Py580 with 1.8 µm of thickness at 20 mM for (**a**) m = ½ and (**b**) m = 2, for band gap energy calculation following Tauc’s Plot.

Direct allowed band gap transition was studied in all thin films doped. The energy band gap is obtained by extrapolating the straight-line portions of the curves to the abscissa axis, as exemplified in Fig. 5 where the ${(\alpha h\nu )}^{1/m}$ vs $h\nu$ for m = 1/2 and m = 2 curves for SU8 doped with Py580 at 20 mM.

Tauc’s model also establishes a graphical way to know if a transition may take place during the absorption process, and that is by showing a straight part in the graph when for a given value m, the relation (αhν)^1/m^ (eV/cm)^1/m^ vs Energy is plotted. In all examinated films, similar graphs were obtained as Fig. 5 shows. Comparable results were previously obtained for dye doped PMMA films^25,26^.

Table 3 summarizes the band gap energies obtained for direct and indirect allowed transitions of all thin films studied, doped and undoped. The calculated values of indirect band gap are lower than the corresponding direct transitions for either doped and undoped thin films.

**Table 3:** Optical band gap obtained for polymeric thin film doped.

Thin film material	Concentration (mM)	${E}_{g}, m=1/2$(eV)	${E}_{g},m=2$(eV)
SU8	–	3.720 ± 0.001	3.560 ± 0.001
EpoCore	–	3.670 ± 0.001	3.520 ± 0.001
OrmoStamp	–	3.690 ± 0.001	3.430 ± 0.001
SU8 + RhB	1	2.126 ± 0.001	2.070 ± 0.001
SU8 + RhB	4	2.117 ± 0.001	2.041 ± 0.001
SU8 + RhB	7	2.115 ± 0.001	2.046 ± 0.001
SU8 + Py-580	1	2.325 ± 0.001	2.291 ± 0.001
SU8 + Py-580	4	2.317 ± 0.001	2.250 ± 0.001
SU8 + Py-580	7	2.318 ± 0.001	2.269 ± 0.001
SU8 + Py-580	20	2.313 ± 0.001	2.248 ± 0.001
EpoCore + RhB	1	2.136 ± 0.001	2.080 ± 0.001
EpoCore + RhB	3	2.130 ± 0.001	2.058 ± 0.001
EpoCore + RhB	5	2.128 ± 0.001	2.094 ± 0.001
OrmoStamp + RhB	2	2.146 ± 0.001	2.086 ± 0.001
OrmoStamp + RhB	5	2.142 ± 0.001	2.069 ± 0.001
OrmoStamp + RhB	7	2.141 ± 0.001	2.066 ± 0.001
OrmoStamp + C540A	30	2.738 ± 0.001	2.520 ± 0.001
OrmoStamp + C540A	50	2.719 ± 0.001	2.495 ± 0.001

The results show that, for the range on concentrations of work in each set of polymers doped, the band gap energy decrease with the concentration. The same phenomenon has been reported by other authors^27^ and it can be attributed to change in the intermolecular interactions of dye molecules amongst themselves and with the polymer molecules.

The generalized increase in bandgap seen in the RhB samples, can be attributed to the same change in the molecular interaction between the dye and polymer matrix compared to a pure thin dye film.

## Discussion

Organic and hybrid organic and inorganic polymeric structures were doped with dyes in order to get self-emitting films and ridge waveguides, and hereby avoiding any need for tedious light coupling into final photonic chip.

For the studies range of dye concentrations, the UV light doses needed to polymerise doped EpoCore, SU8 and OrmoStamp structures has, as expected, been higher than for non-doped resins. The photo-initiated polymerization is hampered by the presence of dye molecules within the matrix. This can be explained because dyes usually have a short tail of absorption within UV range, which means that not all incident UV light will be absorbed by the photoinitiator and because the presence of dye molecules within the material may hamper the molecular cross-linking.

In fact, the concentration limitation in the cases of RhB and Py580 in SU8, was not the solubility of the dye in the matrix, but rather the inhibition of the polymerization. Whether this was caused by an impediment of the polymerization process, or only the photoinitiation has not been studied. If the latter is the cause, it is possible that addition of extra photo-initiator to the prepolymer may allow for higher dye concentrations.

The films doped with RhB show two absorption bands centered at 525 and 565 nm, that correspond to the first π → π^*^ transitions^28,29^. The relative intensities of these bands, and their spectral displacement is a result of quenching and the monomer–dimer equilibrium of dye in the film^30^. The same quenching is also reflected in the emission spectrum of the SU8 doped thin film, where a shoulder at 650 nm is clearly visible.

As expected, the absorption coefficient (α) values increase with the concentration in all doped photoresist. We have achieved higher values of α at maximum of absorption, compared to epoxy-based resins previously reported^31,32^.

Thin films of 4.4 µm of thickness of OrmoStamp doped with RhB showed a considerably low absorption coefficient value compared to epoxy-based resins, even though the thickness of the layer is almost double. It also exhibits a hypsochromic shift with respect to films of SU8 and EpoCore doped with the same dye reflecting that both SU8 and EpoCore are epoxy-based photo-resins while OrmoStamp is a hybrid of organic–inorganic composite, which leads to unalike dye environment and therefore different molecular matrix-dye interactions.

The indirect gap allowed transition range of films doped with RhB is between 2.04 and 2.09 eV. These values are a bit greater than previously reported RhB thin films band gaps^33^. This small discrepancy might be attributed to the fact that RhB molecules are not only interacting with each other but also with the polymer molecules, which leads to an increase in the potential experienced by the electrons. This is analogous to the temperature dependence of band gap in organic polymers films^34^.

Band gap values for direct transitions of undoped polymers are in the range of 3.69–3.72 eV.

The resulting waveguide emitters provide a light source that at the time of manufacturing can be integrated in a more complex waveguide sensor. The emitters may be excited with an external light source that need not to be aligned in order to get an adequate coupling of the relevant light wave. Hence the implementation of such a said device becomes trivial. Having developed the emitters in polymers, compatible with other polymer approaches, have the potential of future plastic-based roll-to-roll development of optical sensors of a vastly reduced complexity when compared to other, similar, technologies.

## Conclusions

Thin films and micro structured waveguides of photoresist as EpoCore, OrmoStamp and SU8 with different dye content, covering the visible range, have been manufactured and studied. The dyes/matrix combinations which provided good results were RhB/SU8, RhB/Epocore, Py580/SU8 and C540A/Ormostamp. Absorption coefficients, α, were obtained from absorption spectra of all doped thin film. The emission spectra of the samples were also recorded. The band gap energy of direct and indirect allowed transitions, of the dyes within the solid matrices at different content and undoped resists were obtained by using Tauc’s model. The high band gap energy for undoped resists (3.69–3.72 eV for direct and 3.43–3.56 eV for indirect transitions) shows their excellent optical transparency at visible light. Thin films of dye doped photoresists in this study might be used in photonic integrated circuits, in photonic lab-on-chip application or even as base material for the creation of lasing and amplifying devices.

## Supplementary Information

Supplementary Information.

## Data Availability

All data generated or analyzed during this study are included in this published article (and its Supplementary Information files).
