# Supplementary material for: Identification of dyes and matrices for dye doped polymer waveguide emitters covering the visible spectrum
Source: Sci Rep. 2022 Apr 12;12:6142. doi: 10.1038/s41598-022-10145-8 (PMC9005527; doi:10.1038/s41598-022-10145-8)
Supplement: Supplementary file 1 — Supplementary Information. [file 41598_2022_10145_MOESM1_ESM.pdf]

Supplementary information

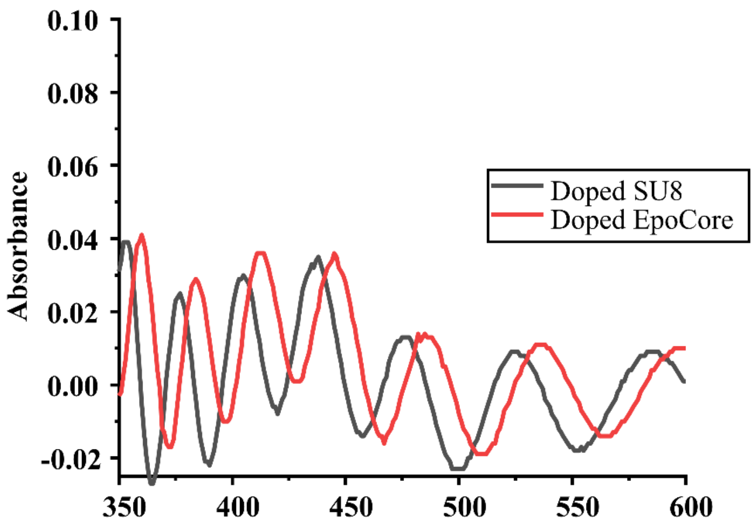

S1. Absorption spectra of polymerized thin films of SU8 and EpoCore doped with C540A at 5 mM with thickness of and 1.8 and 2 µm, respectively.

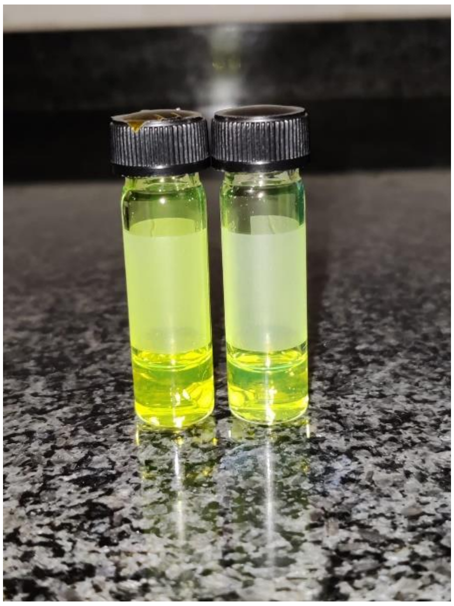

S2. Prepolymer solutions of EpoCore (left) and SU8 (right) doped with 5 mM of C540A.

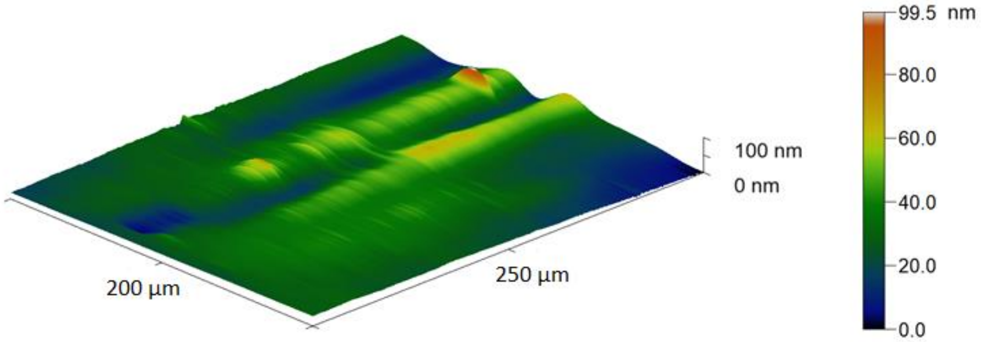

S2. 3D m.

S3. 3D Surface topography of thin film of SU8 doped with RhB at 7 mM.

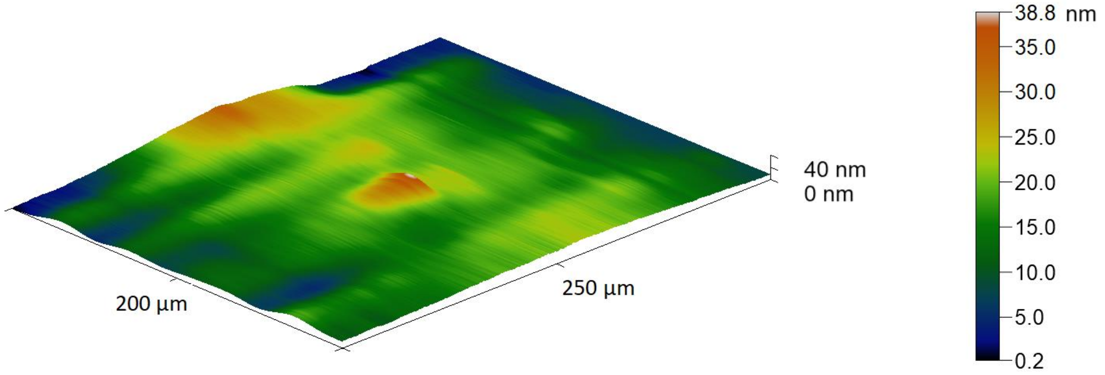

S4. 3D Surface topography of thin film of EpoCore doped with RhB at 5 mM.

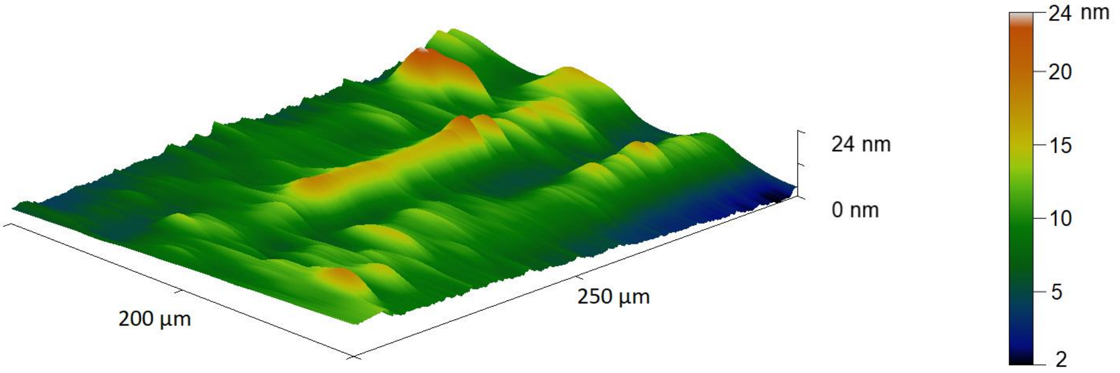

S5. 3D Surface topography of thin film of OrmoStamp doped with RhB at 7 mM.

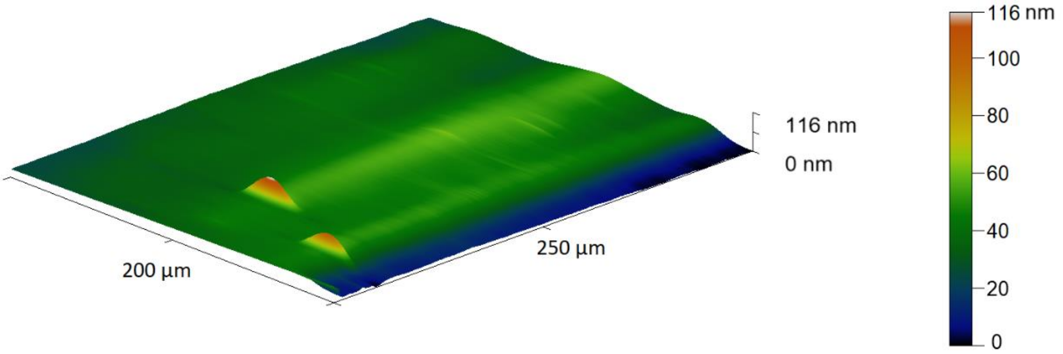

S6. 3D Surface topography of thin film of SU8 doped with Py580 at 20 mM.

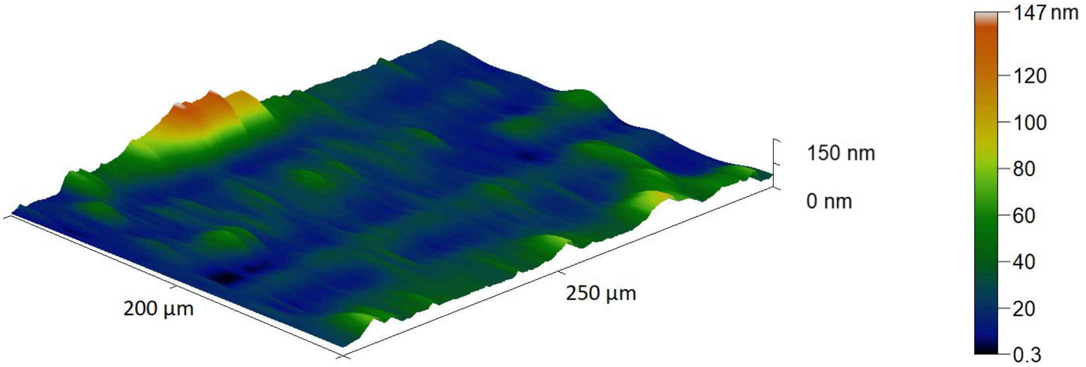

S7. 3D Surface topography of thin film of OrmoStamp doped with C540A at 50 mM.

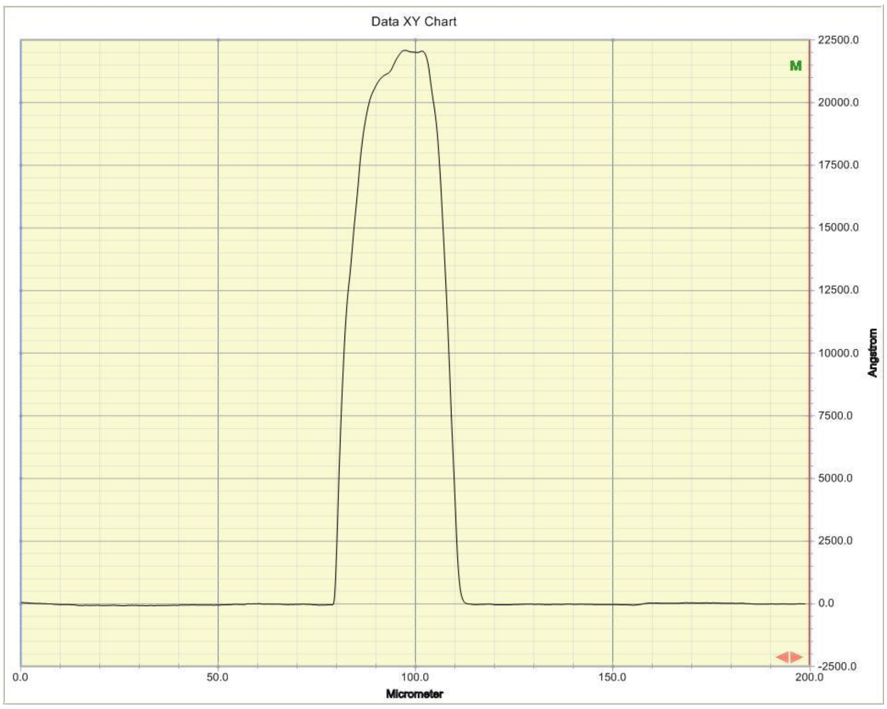

S8. Profile of waveguide of EpoCore doped with Rhb.

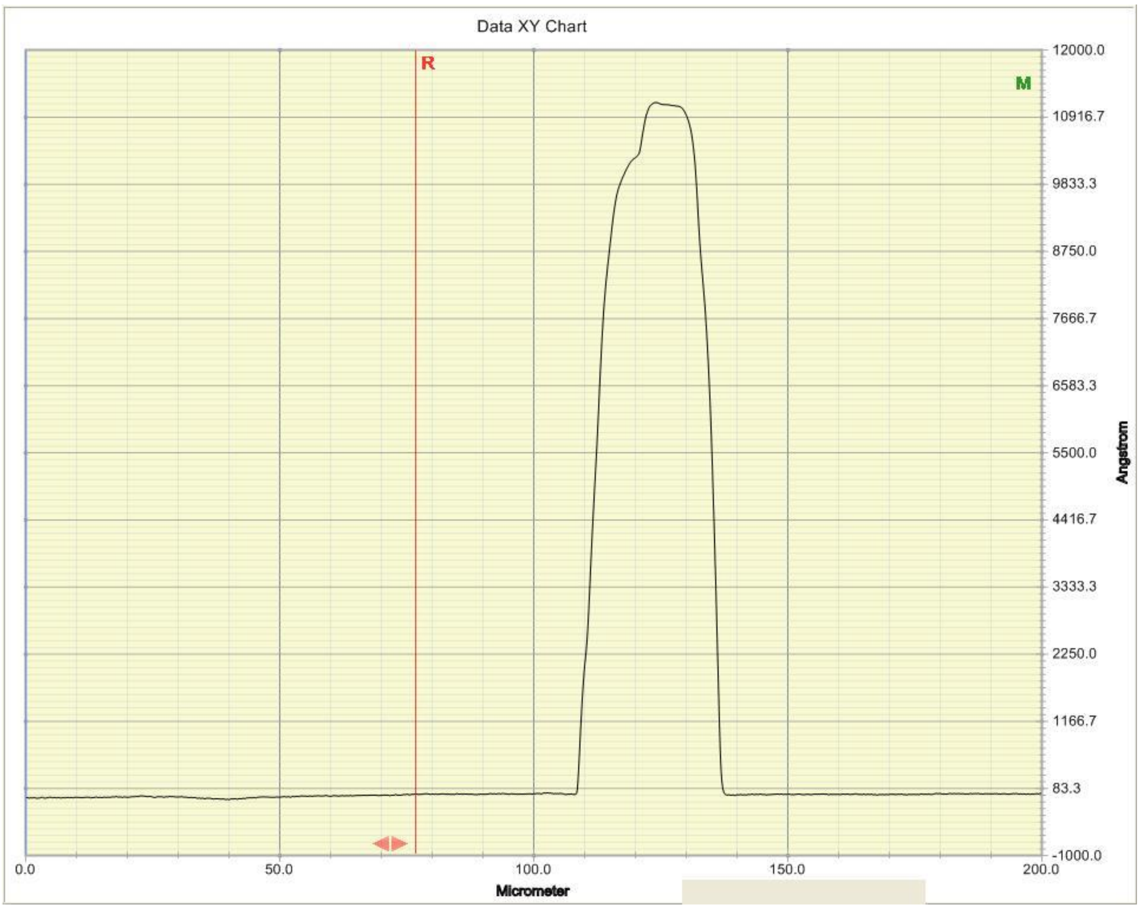

S9. Profile of waveguide of SU8 doped with Rhb.

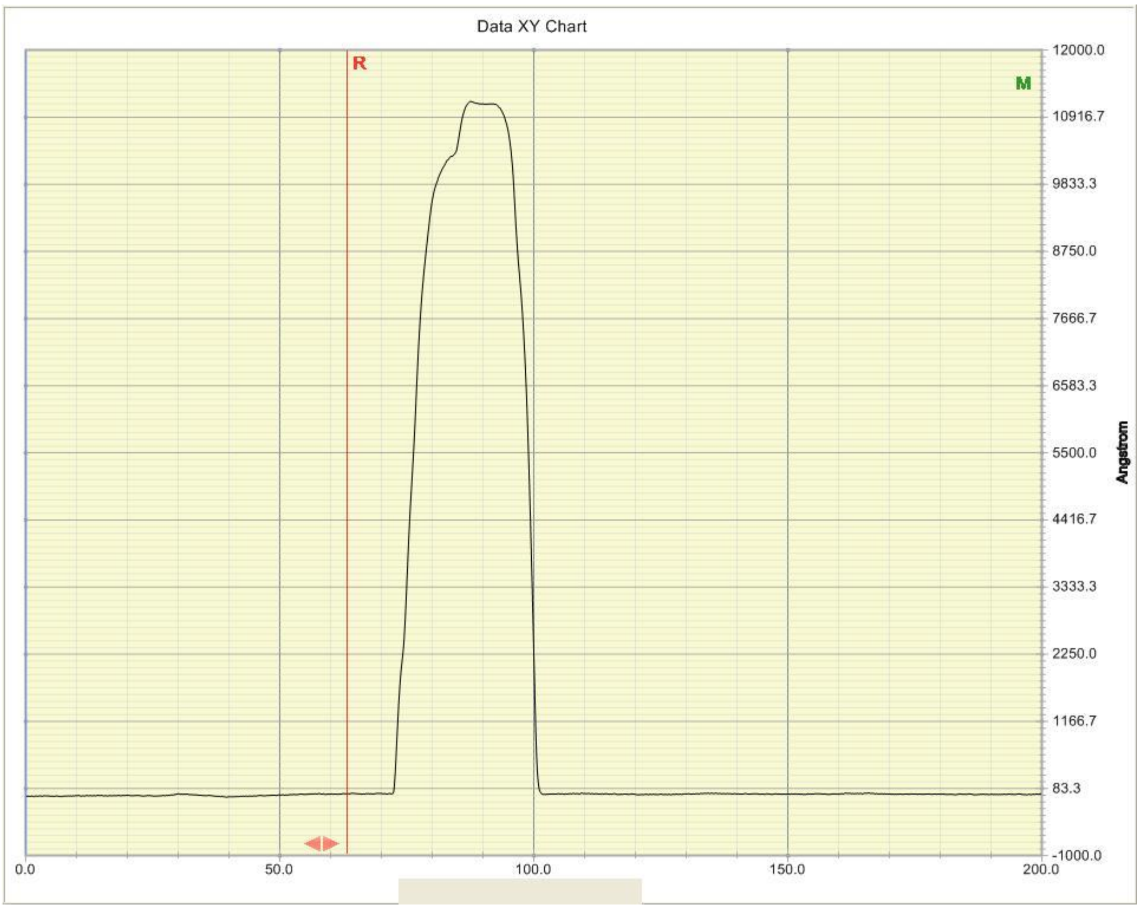

S10. Profile of waveguide of SU8 doped with Py580.

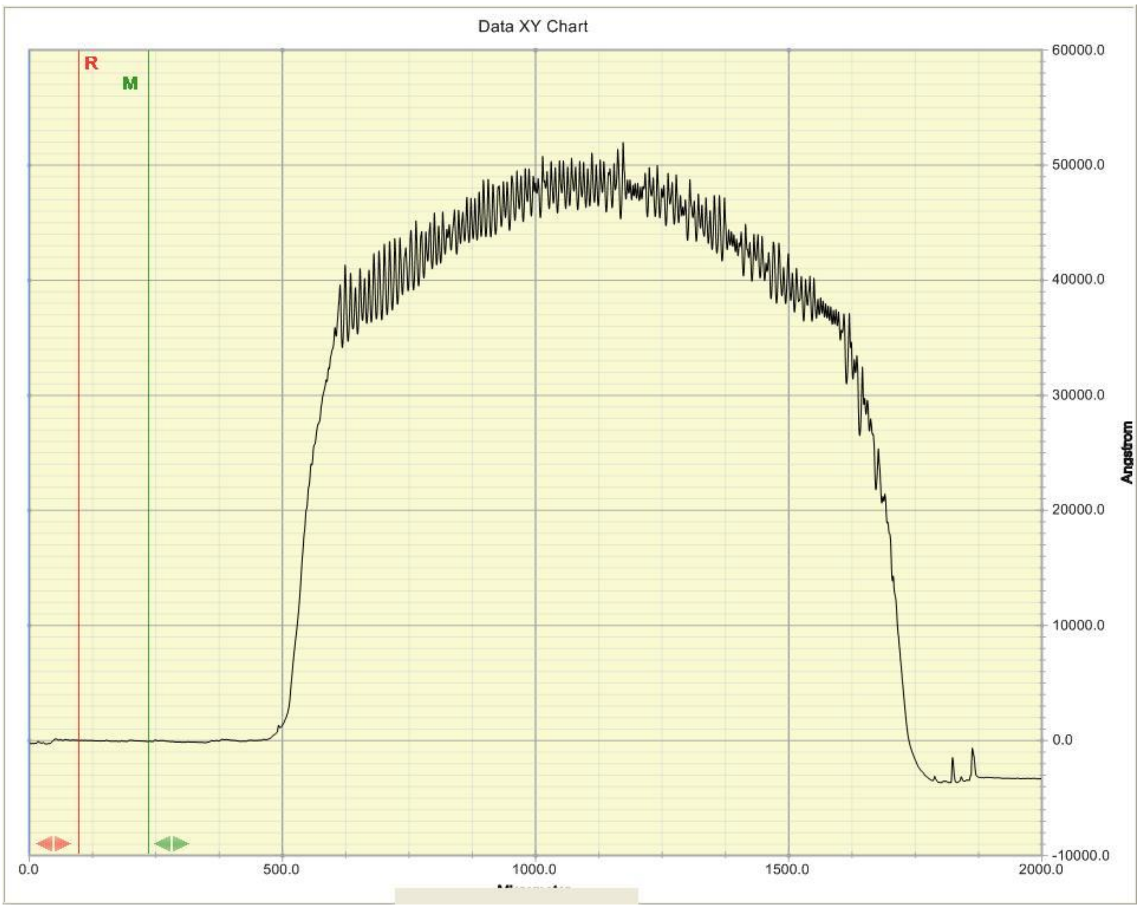

S11. Profile of waveguide of OrmoStamp doped with Rhb.

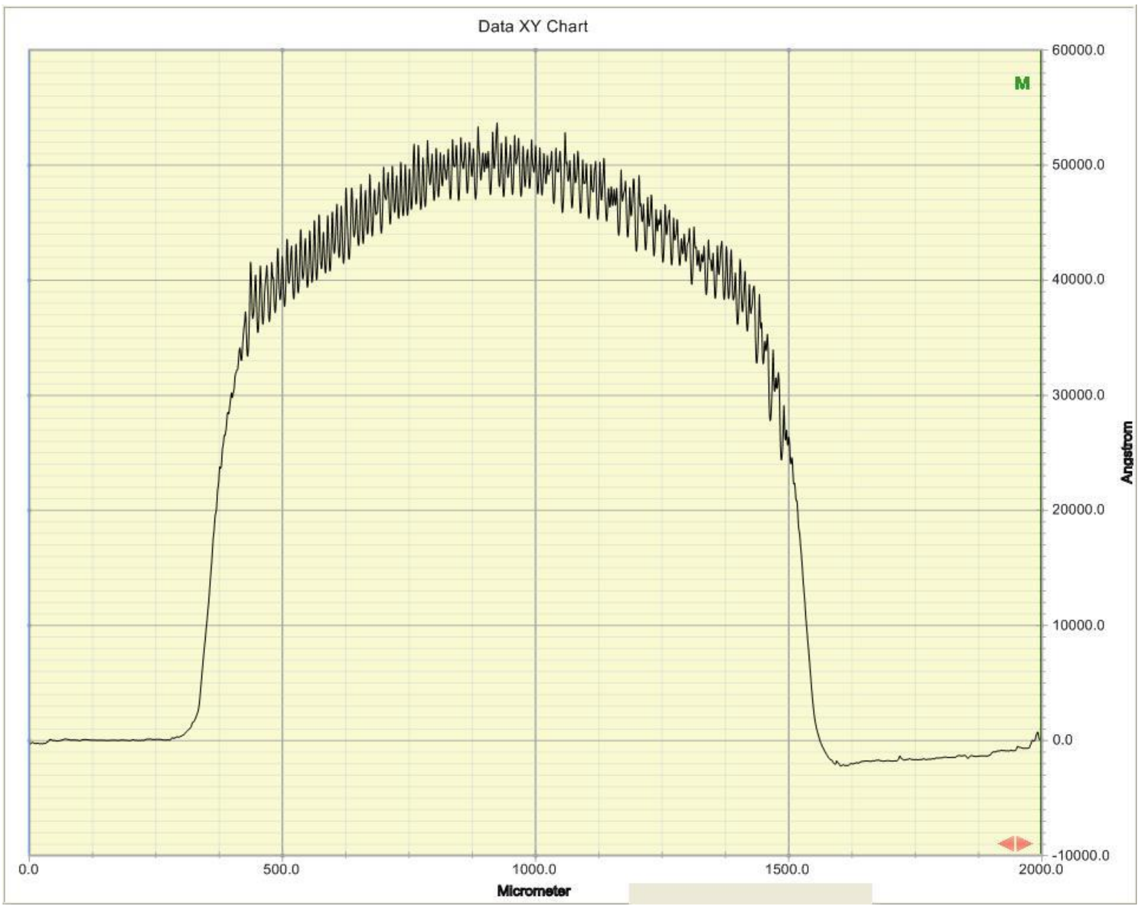

S12. Profile of waveguide of OrmoStamp doped with C540A.

Table S1. Maximum absorption coefficients values (α) of all doped resists under study.

	1 mM	2 mM	3 mM	4 mM	5 mM	7 mM	10 mM	20 mM	30 mM	50 mM
**SU8-RhB**	870.02			3249.8		5629.6				
**EpoCore-RhB**	826.03		2263.1		3569.7					
**OrmoStamp-RhB**	519.9				942.1	1481.2				
**SU8-Py580**	637.62			2379.8		3620.8		9954.1		
**OrmoStamp-C540A**							-		471.1	900.3
